# Tracking down the sources of experimental contamination in microbiome studies

**DOI:** 10.1186/s13059-014-0564-2

**Published:** 2014-12-17

**Authors:** Sophie Weiss, Amnon Amir, Embriette R Hyde, Jessica L Metcalf, Se Jin Song, Rob Knight

**Affiliations:** Department of Chemical and Biological Engineering, University of Colorado at Boulder, Boulder, CO 80309 USA; BioFrontiers Institute, University of Colorado at Boulder, Boulder, CO 80309 USA; Department of Chemistry and Biochemistry, University of Colorado at Boulder, Boulder, CO 80309 USA; Howard Hughes Medical Institute, Boulder, CO 80309 USA

## Abstract

A recent report warns that DNA extraction kits and other laboratory reagents are considerable sources of contamination in microbiome experiments. The issue of contamination is particularly problematic for samples of low biomass.

See related research, http://www.biomedcentral.com/1741-7007/12/87

High-throughput sequencing has revolutionized our understanding of the microbial world, providing a means by which we can characterize microbial communities in considerable detail without being affected by biases introduced by culture-based protocols that might reveal only a small fraction of the community. We have learned that, although humans share over 99.9% of their genomic DNA sequence with one another, they might share as little as 10% of their microbes at a given body site. Therefore, an intriguing hypothesis is that some aspects of the human phenotype might be determined more by microbial DNA than human DNA. Over the past five years, an enormous push in microbiome research has elucidated many of the factors that can affect this microbial individuality – the human microbiome is affected by diet, culture, geography, age and antibiotic use, among other factors [1]. Importantly, the microbiome has been implicated in numerous health conditions through correlative studies in humans and experimental research in mouse models. These conditions range from obesity [2] to multiple sclerosis [3]. However, if samples are not collected, processed, and analyzed properly, this may lead to erroneous conclusions.

## Microbes are increasingly studied in low-biomass environments

Microbes play crucial roles not just in human-associated ecosystems – they are ubiquitous in every environment, from deep ocean vents to the arctic. However, this ubiquity also poses major challenges in controlling for background contamination present in the air, laboratory surfaces, the skin and clothing of researchers, and in laboratory reagents. In the November issue of *BMC Biology*, Salter and colleagues [4] present a comprehensive study of contaminant sources in microbiome experiments and demonstrate the great influence that contamination can have on readouts of microbial communities based on DNA. These effects are especially important in studies focusing on samples of low biomass.

Much of recent high-impact microbiome research has focused on the gut, which is characterized using fecal samples as a proxy for the distal large intestine. Fecal samples have such high biomass that the DNA of fecal microbes almost certainly overwhelms contaminating background microbial DNA from reagents and other sources. However, as microbiome research expands in scope to include samples of lower biomass, such as the airways, placenta or even blood plasma, the standard high-throughput approaches often used for fecal samples will probably not be sufficient to generate reliable readouts of the microbial communities or assemblages associated with such samples. This problem arises because, as the ‘true’ biomass becomes smaller, the potential for contaminants occupying a larger fraction of the sequences will become greater. For example, a recent study by Kennedy and colleagues [5] showed that PCR template concentration, which is associated with sample biomass (especially when extracted DNA concentrations are not normalized before downstream processing, which is common in high-throughput settings), significantly affects the resulting microbial community profile.

## Sample contamination can come from many sources

Several sources can contribute to sample contamination and can occur at several steps, occurring between collection and sequencing. The use of non-sterile equipment, or accidental exposure to the environment or researcher, can contaminate the sample. However, it should be noted that microbial DNA can be present even in sterile equipment. Therefore, strict protocols, such as the use of clean-suits, gloves, facemasks, and bleach and UV for cleaning equipment, could be needed to prevent contamination during sample collection. Microbial DNA can also be introduced during sample processing, either during initial microbial DNA extraction or during PCR amplification, in the case of marker gene amplification and sequencing (multiple displacement amplification (MDA) and related techniques can also amplify reagent contaminants during library preparation for shotgun metagenomic sequencing). In reality, microbial DNA that is not endogenous to the samples being studied probably contaminates every microbiome dataset to some extent. The work by Salter *et al*. [4] takes important steps in helping us to determine what these contaminants are, where they come from and how large an effect they can have on research results.

To investigate the diversity of microbial contaminants, the researchers used an elegant combination of positive and negative (blank) controls. They used a pure culture of *Salmonella bongori*, which has not been observed as a common contaminant, in a series of five 10-fold dilutions to assess the effect of background contamination on samples with varying biomass [4]. Using 16S ribosomal RNA (rRNA) gene amplification and high-throughput sequencing, along with typical PCR-amplified ‘blank’ controls comprising ultrapure water, they distinguished contaminants arising from DNA extraction kits and other sources, including PCR kit reagents, laboratory consumables and personnel. Salter and colleagues [4] show very clearly that contaminating organisms became increasingly dominant as the biomass of *S. bongori* decreased, with contaminants representing the majority of the microbial biomass by the fifth dilution.

Sixty three taxa were unique to the diluted samples compared with the PCR ‘blank’ control, implicating the DNA extraction kit as a likely contaminant source. Salter and colleagues also analyzed metagenomes produced through shotgun sequencing of non-amplified bacterial DNA, which, unlike the 16S rRNA gene-sequencing protocol, does not include a targeted PCR step and thus eliminates the introduction of contamination through PCR. Nonetheless, the authors observed similar results, with contaminants dominating in low-biomass samples, and again implicating the DNA extraction kit as the source of contaminants [4]. Interestingly, of the four DNA extraction kits that Salter *et al.* tested, the lowest levels of contamination appeared to result from the use of the MoBio kit, which is the kit used by most of the major microbiome studies, such as the Human Microbiome Project (http://www.hmpdacc.org/) and Earth Microbiome Project (http://www.earthmicrobiome.org/).

## Contamination can affect biological conclusions, especially when confounded with other variables

Salter and colleagues [4] then demonstrated how contamination could affect interpretation of biological studies by analyzing low-biomass samples from a recent study of nasopharyngeal microbes during infant development [6]. The authors found that, in the original dataset, contaminant operational taxonomic units (OTUs) associated with different batches of the same extraction kit drove the clustering patterns found in principal coordinate analysis (PCoA) space, which led to the misleading conclusion that the composition of the nasopharyngeal microbiome changed with age. Once contaminant OTUs were removed from the dataset and the primary samples were reprocessed using a different extraction kit, samples no longer clustered by age, thereby significantly altering the research results and interpretation [4].

Such batch effects have already been observed in genomic data [7]. As suggested by Leek and colleagues, a good way to check that an experimental, rather than biological, variable is driving the PCoA clustering is to test whether the experimental variable correlates strongly with the major principal components. This procedure assumes that the samples have been randomly assigned to DNA extraction batches, PCR batches and DNA sequencing-instrument runs: a common mistake, which should clearly be avoided, is to confound experimental variables (such as time-point) or clinical variables (such as case versus control status) with one or more of these variables, making resolution of the biological effect against the background of these technical effects in principle impossible. OTU-based analyses, such as correlation networks or differential-abundance testing, are even more sensitive to any type of contaminant. This sensitivity arises because each sample has a constrained total number of sequences; therefore, any change in one OTU affects all others in that sample. Furthermore, any taxa that are present in the blanks should be monitored carefully during the rest of the analysis, as recommended by Salter *et al*. [4].

The implications of this study are that microbiome researchers might need to take additional precautions in the laboratory and develop both laboratory and bioinformatics workflows for monitoring contamination. As part of their conclusion, the authors recommend a reasonable set of steps for minimizing the effects of contaminants before, during and following sequencing, including the use of negative controls, technical replicates, sample randomization and keeping records of kits and other reagents [4]. However, this study also highlights the need for additional studies that benchmark methods and protocols in microbiome research. For example, researchers might want to consider using different concentrations of a single bacterial culture as a control, which could produce better estimates of the degree and nature of contamination than reagent blanks.

## Concluding remarks

Owing to the high sensitivity of high-throughput sequencing-based microbiome analysis, reproducibility (how well the results repeat themselves) and bias (how well the results reflect the reality) can be a major concern. The work of Salter and colleagues [4] is a springboard from which microbiome researchers, who have been controlling for contamination primarily within individual labs, can begin to build a consensus for laboratory and bioinformatics approaches, thus helping researchers avoid spurious results and saving valuable money, time and effort. This work builds on previous studies [8–10], and recently the microbiome quality-control project (http://www.mbqc.org/), that rigorously tested variability introduced by differences in methodology, such as storage, preservation, extraction and analysis, and, especially, highlights taxa that might systematically point to reagent contamination [8]. However, contamination from other biological sources, and especially the mouth and skin of the investigators conducting the studies, should also be considered as a possibility when reviewing results that are surprising in the light of prior knowledge of the biological niches of the organisms involved. Together, all these efforts are beginning to close important gaps of knowledge in microbiome research and provide essential resources that inform better study design and practices for all microbiome researchers.

## Abbreviations

MDA: Multiple displacement amplification
OTU: Operational taxonomic unit
PCoA: Principal coordinates analysis
PCR: Polymerase chain reaction
rRNA: Ribosomal RNA
